# Supplementary material for: Modelling the effect of V1a receptor antagonism and its potential therapeutic effect in circadian disorders
Source: NPJ Biol Timing Sleep. 2026 Jun 3;3:23. doi: 10.1038/s44323-026-00085-1 (PMC13234031; doi:10.1038/s44323-026-00085-1)
Supplement: Supplementary file 1 — Supplementary Material [file 44323_2026_85_MOESM1_ESM.pdf]

Modelling the effect of V1a receptor antagonism and its potential therapeutic effect in circadian disorders

Supplementary material

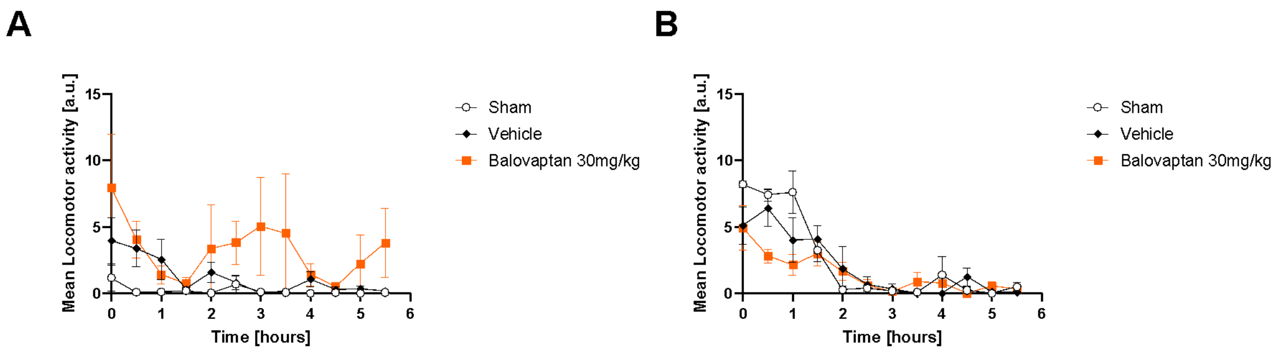

Figure S1: *locomotor activity of mice in bins of 30 minutes (Mean and SEM) (A) first experiment (B) second experiment.*

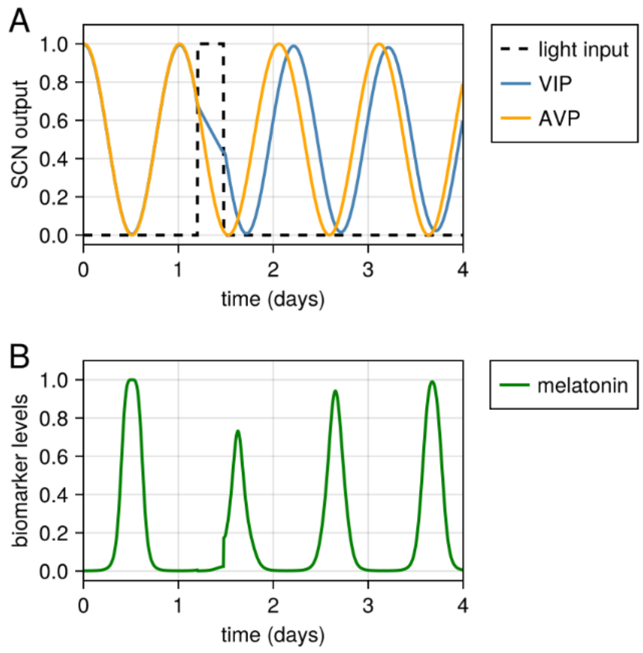

Figure S2. Impact of a light pulse on SCN output and melatonin levels. *This figure shows the effect of a light pulse administered before the expected melatonin rise on A) SCN output and B) melatonin levels. The model accurately replicates the suppression of melatonin onset and the resulting in a slightly shorter consequent melatonin peak, aligning with experimental findings.*

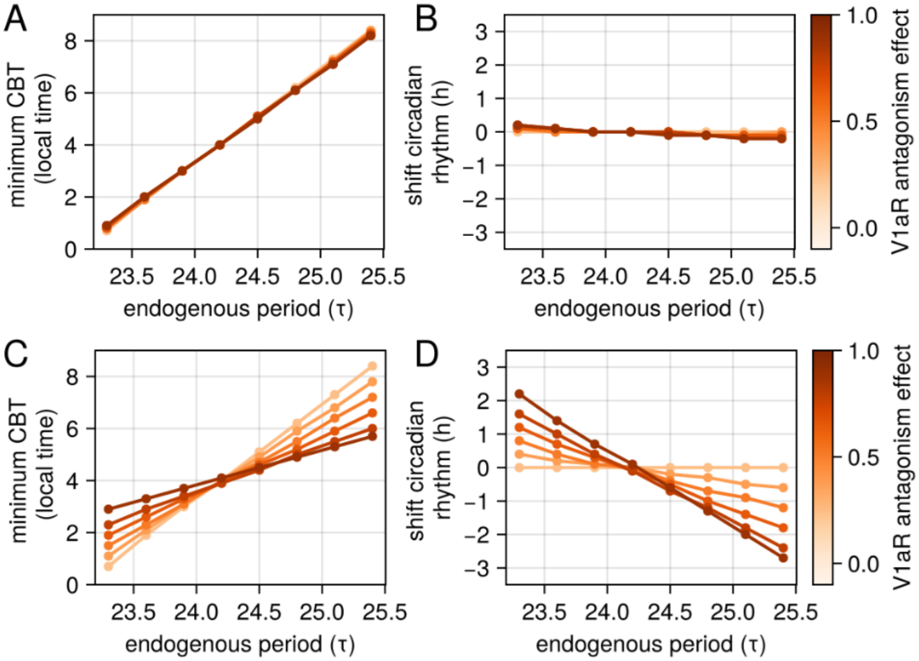

Figure S3. *A,B) same as figure 7E,F with V1aR antagonism effect only acting on* κ_{AV}*. C,D) same as figure 7E,F with V1aR effect only on* κ_{AA}*.*
